# Methylprednisolone-Induced Delayed and Sustained Bradycardia in Multisystem Inflammatory Syndrome in Children

**DOI:** 10.7759/cureus.107919

**Published:** 2026-04-28

**Authors:** Sujin M Thomas, Jijo J John, Keerthy Kurian, Alice David, Aby Dany Varghese, Midhuna TV, Jomon V Thomas

**Affiliations:** 1 Pediatric Medicine, Believers Church Medical College, Thiruvalla, IND; 2 Pediatric Medicine, Believers Church Medical College Hospital, Thiruvalla, IND; 3 Medical Research, Believers Church Medical College Hospital, Thiruvalla, IND; 4 Pediatric Medicine, Balagangadharanatha Swamiji (BGS) Medical College and Hospital, Adichunchanagiri University, Bangalore, IND

**Keywords:** bradycardia, covid, heart rate, methylprednisolone, multisystem inflammatory syndrome in children

## Abstract

Background: Therapeutic immunomodulation using pulse methylprednisolone is one of the cornerstones of management in multisystem inflammatory syndrome in children (MIS-C). This study examines whether bradycardia was possibly methylprednisolone-induced and if the risk and time taken to develop or recover from bradycardia depend on the dose or presence of shock.

Methods: A longitudinal study of those with MIS-C who received methylprednisolone treatment was conducted. Baseline heart rate (HR) prior to starting treatment was documented, and heart rate was monitored every two hours by bedside cardiac monitors. Student's t-test was used to test the difference in means after log transformation if required. Kaplan-Meier curves were used to estimate the median time to onset and recovery of bradycardia, and Mood's two-sample test was used to test the difference in medians.

Results: Out of 89 children with MIS-C included in the study, most (84.3%) developed bradycardia after methylprednisolone administration, with a significant drop in heart rate irrespective of age. The median time of onset was 31.0 (interquartile range (IQR): 10.0-80.0) hours after the administration of methylprednisolone, and recovery occurred 57.0 (IQR: 14.0-202.0) hours later. This was not significantly different by dose or by the presence of shock. Although the proportion of bradycardia were similar across groups, the heart rate was significantly lower at bradycardia for those on pulse dose and those with shock when compared to those on conventional dose and those who did not have shock, respectively.

Conclusion: A high proportion of delayed and sustained bradycardia in children with MIS-C, irrespective of age, dose of methylprednisolone, or presence of shock, which is possibly induced by the steroid, was observed. The time to onset and the time to recovery were independent of the above factors. However, the heart rate at bradycardia varied significantly by dose and by the presence of shock.

## Introduction

The immunomodulatory benefits of methylprednisolone are well-documented. However, its cardiac side effects warrant closer clinical scrutiny. Although traditionally considered rare, bradycardia is a highly prevalent adverse event of corticosteroid therapy in two specific pediatric conditions, based on available literature, one being acute lymphoblastic leukemia (ALL) and the other being severe Kawasaki disease (KD). That is, children with acute lymphoblastic leukemia (ALL) receiving induction chemotherapy develop steroid-induced bradycardia in up to 98% of cases [1], and the prevalence of bradycardia was 79.1% in children treated with intravenous immunoglobulin (IVIG) plus prednisolone. This was much higher than that of those receiving IVIG alone, for whom the prevalence was only 7.1% [2].

Since the underlying pathophysiology of multisystem inflammatory syndrome (MIS-C), a rare but life-threatening hyper-inflammatory condition temporally associated with SARS-CoV-2 infection, occurring predominantly in the pediatric population [3], involves a dysregulated immune response characterized by a "cytokine storm," with excessive activation of T cells and pro-inflammatory cytokines, including interleukin-6 (IL-6), IL-10, and tumor necrosis factor-alpha (TNF-α), therapeutic immunomodulation is the cornerstone of management to mitigate this hyper-inflammatory state and prevent irreversible myocardial damage. Therefore, intravenous methylprednisolone, a potent glucocorticoid, was the primary treatment modality used to suppress cytokine transcription and stabilize the vascular endothelium [4,5]. It was administered in conventional doses (1-2 mg/kg/day) for moderate cases or pulse doses (10-30 mg/kg/day) for severe, refractory disease or those presenting with hemodynamic shock [6].

We observed that bradycardia was highly prevalent in our study subjects and therefore propose that MIS-C is a third condition where methylprednisolone-induced bradycardia is highly prevalent. As per available literature, a case report [7] and a few case series with a maximum of 46 cases have suggested that the effect of methylprednisolone could have possibly caused bradycardia [8,9]. Efforts have been made, in this study of 89 patients, to establish the same using the WHO-adverse drug reaction (ADR) causality assessment scale [10] rather than withdrawing treatment or having another comparative group, for ethical reasons. That is, the lack of an alternative treatment for MIS-C prevented us from pursuing the traditional method of causality. Additionally, a dose response would add to the evidence of causality of drug-induced ADR.

Moreover, given that cardiac involvement is a defining hallmark of MIS-C, occurring in up to 80% of cases [11], which may manifest as acute myocarditis, coronary artery dilatation, or arrhythmias, we propose that the bradycardia observed in these children is not a manifestation of the disease but an adverse effect of the treatment given. Clinicians encounter significant challenges in differentiating benign, drug-induced bradycardia from disease-related cardiac deterioration. Misattributing this pharmacological effect to the underlying disease may lead to unnecessary diagnostic escalation, such as repeated echocardiography and 12-lead electrocardiograms (ECG), extended intensive care unit (ICU) admissions, and the avoidance of essential steroid therapy, thus highlighting the relevance of this study.

Moreover, since recent evidence indicates that baseline hemodynamic shock serves as an independent predictor of subacute myocardial dysfunction during follow-up, it is clinically imperative to ascertain whether it also exacerbates the bradycardic response. Specifically, our objectives are to ascertain the incidence of bradycardia in MIS-C, investigate whether the heart rate (HR) varies by steroid dose or the presence of shock at various time points, and to estimate the average time taken for onset and recovery. The latter two objectives have not been studied in any of the previous studies.

## Materials and methods

This longitudinal study was undertaken by reviewing records of children who were diagnosed with MIS-C as per the WHO case definition [12] between October 2021 and June 2022 in a tertiary care hospital in central Kerala, India, after obtaining approval from the Institutional Research and Ethics Committee (date: 23.05.2024, IEC study number: IEC/2024/08/421). Standard protocol-based definitions were used for all clinical parameters in this study. Hemodynamic shock was defined as per the Advanced Paediatric Life Support (APLS) guidelines [13]. Bradycardia was defined as a heart rate less than the fifth percentile for age, as defined by APLS guidelines.

All children with MIS-C who received methylprednisolone were included. The conventional dose of methylprednisolone used is 2 mg/kg/day. However, some children received a high dose of 30 mg/kg/day (pulse dose) intravenously. The dose depended on the child's clinical condition. Children who satisfied the definition of MIS-C and had features of shock, life-threatening disease, organ failure, or dysfunction received pulse dose, and the rest of them received conventional dose. However, those with prior steroid exposure within four weeks of diagnosis and children with underlying cardiac conditions were excluded.

The children included in the study were monitored closely with bedside cardiac monitors. The baseline heart rate before starting treatment was documented, and the heart rate was continuously monitored using bedside monitors. Alarm limit was set to identify any deviation from the normal range. The second hourly heart rate was manually recorded by the staff nurse in the patients' charts. Any deviation from the normal range was also recorded in the patients' vitals charts. The time of development of bradycardia was obtained from the monitoring sheet. Post-discharge follow-up was conducted at one week, two weeks, and one month. At each outpatient visit, heart rate was recorded by auscultation and pulse oximetry by the treating pediatrician. Children with persistent bradycardia at the first review were scheduled for additional follow-up visits until resolution was confirmed.

Statistical methods

Baseline characteristics were assessed using descriptive statistics. Comparison of proportions of bradycardia was done using the chi-square test. Continuous variables were tested for normality using the Q-Q plot. If not normal, transformation was done either by removing single outliers or using log transformation. Student's t-test was used to compare means of transformed data. A p-value of <0.05 was considered significant. The median time taken to the onset of bradycardia or recovery from bradycardia was estimated using Kaplan-Meier curves. The comparison of each of time taken mentioned above between conventional and pulse dose and between those with and without shock was done using Mood's median test. Complete heart rate data at all time points were available for all 89 included patients. As time-to-event data (time to onset and recovery from bradycardia) were non-normally distributed with substantial right skew and censoring (children discharged before bradycardia resolution), Kaplan-Meier estimation was used. Patients discharged before resolution of bradycardia was treated as censored observations. The comparison of each of time taken mentioned above between conventional and pulse dose and between those with and without shock was done using Mood's median test.

All statistical analyses, including graphs, were performed using SAS version 9.4 (SAS Inc., Cary, NC) [14].

## Results

**Figure 1:**
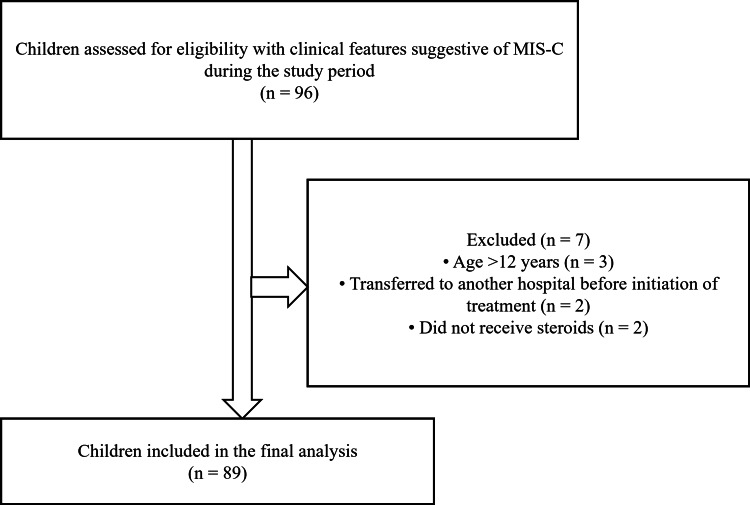
Flowchart illustrating the selection of the study cohort The diagram depicts the identification and selection process of children treated with steroids for MIS-C. A total of 96 medical records of children presenting with clinical features suggestive of MIS-C during the study period were reviewed. Seven cases were excluded from the final analysis: 3 patients were over 12 years of age, 2 were transferred to another facility prior to the initiation of treatment, and 2 patients did not receive steroid therapy. The final analysis was conducted on a cohort of 89 children who fulfilled the inclusion criteria. MIS-C: multisystem inflammatory syndrome in children

A total of 96 children were diagnosed with multisystem inflammatory syndrome in children (MIS‑C) during the study period, of whom 89 met the inclusion criteria and were included in the final analysis, as shown in Figure 1.

Baseline demographic and clinical characteristics of the study population are summarized in Table 1.

**Table 1:** Baseline characteristics of children with MIS‑C treated with methylprednisolone (N = 89) Data are presented as mean ± SD with range or number (%). Bradycardia was defined as a heart rate <5th percentile for age (APLS guidelines). SD: standard deviation, APLS: Advanced Paediatric Life Support, ICU: intensive care unit

Patient characteristics	N = 89
Age in years (mean ± SD (range))	4.1 ± 3.2 (0.2-11.7)
Female (number (%))	41 (46.1%)
Duration of hospital stay in days (mean ± SD (range))	4.8 ± 3.5 (1-20)
Duration of ICU stay in days (mean ± SD (range))	3.9 ± 2.6 (0-19)
Shock (number (%))	16 (18.0%)
Conventional dose (number (%))	52 (58.4%)
Bradycardia by age (number (%))	
0-1 years (n = 15)	13 (86.7%)
1-2 years (n = 18)	15 (83.3%)
2-5 years (n = 27)	23 (85.2%)
5-12 years (n = 29)	24 (82.8%)
Total	75 (84.3%)

Heart rate values at admission, immediately prior to initiation of methylprednisolone therapy, during hospitalization, and at discharge were recorded, as shown in Table 2.

**Table 2:** Distribution of heart rate at various time points by age Data are presented as mean ± SD. Comparisons of means between heart rate at admission and heart rate at bradycardia were performed using Student's t-test. Test statistic values represent t-values. Statistical significance was at the following levels: *p < 0.01, **p < 0.0001, and *** p < 0.001 ^#^Not significant SD: standard deviation

Age	Number	Normal range	I. Heart rate at admission (mean ± SD)	II. Heart rate immediately after steroid (mean ± SD)	|t| value, I versus II	III. Heart rate at bradycardia (mean ± SD)	|t| value, II versus III	IV. Heart rate at discharge (mean ± SD)	|t| value, III versus IV
0-1	13	110-160	149.9 ± 18.7	132.8 ± 10.8	3.1*	94.7 ± 9.8	9.7**	109.9 ± 8.5	4.4***
1-2	15	100-150	129.7 ±13.6	124.2 ±13.3	1.2^#^	90.8 ± 6.3	8.9**	107.4 ± 15.0	4.0***
2-5	23	95-140	133.4 ± 23.8	117.3 ± 18.7	2.8*	75.2 ± 9.5	9.8**	92.3 ± 21.6	3.5***
5-12	24	80-120	127.3 ± 21.2	110.4 ± 21.5	3.0*	68.8 ± 8.6	8.9**	92.8 ± 12.4	8.0***

**Figure 2:**
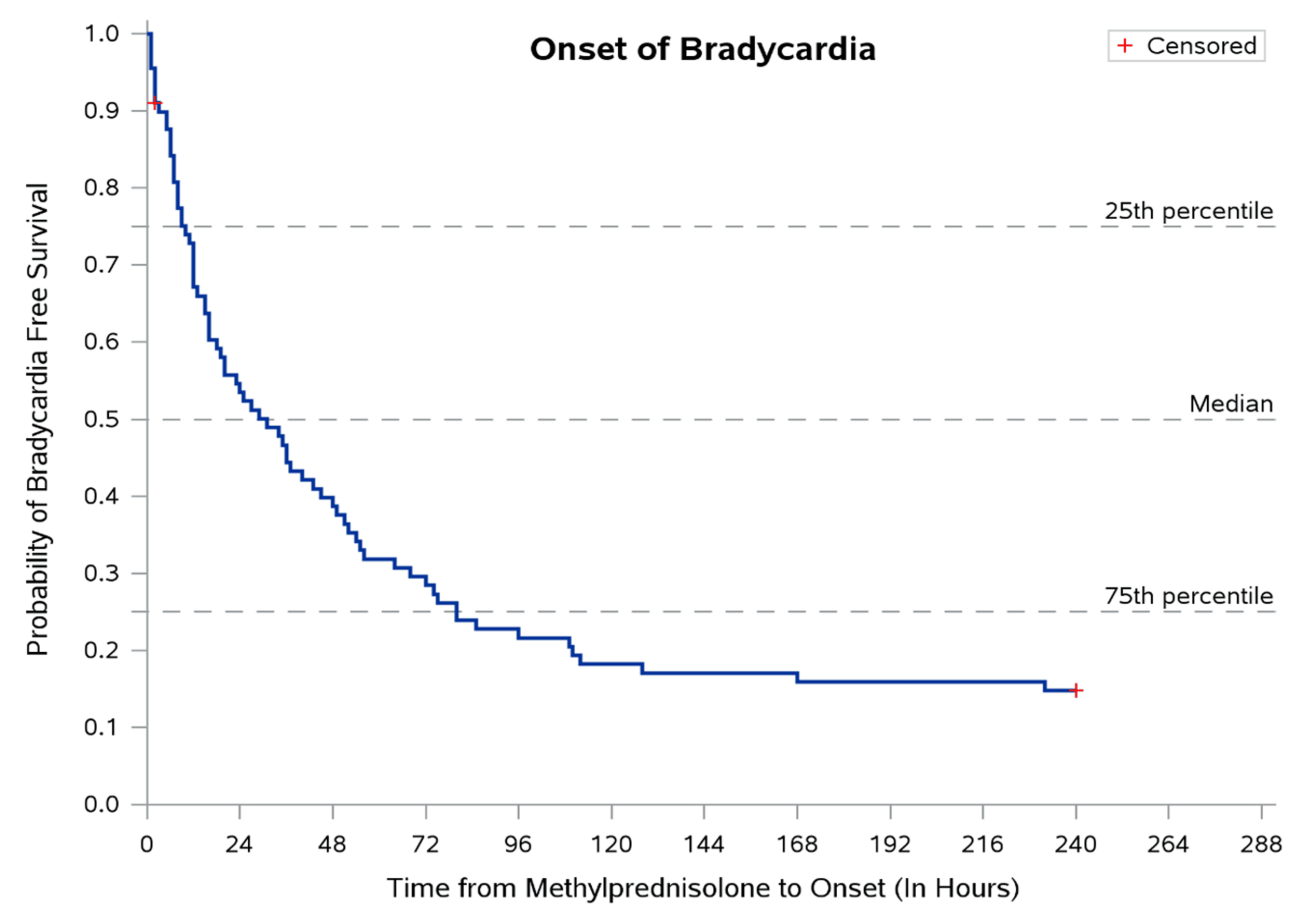
Time to onset of bradycardia following steroid administration Median time to onset of bradycardia following steroid administration. Error bars represent the IQR. IQR: interquartile range

**Figure 3:**
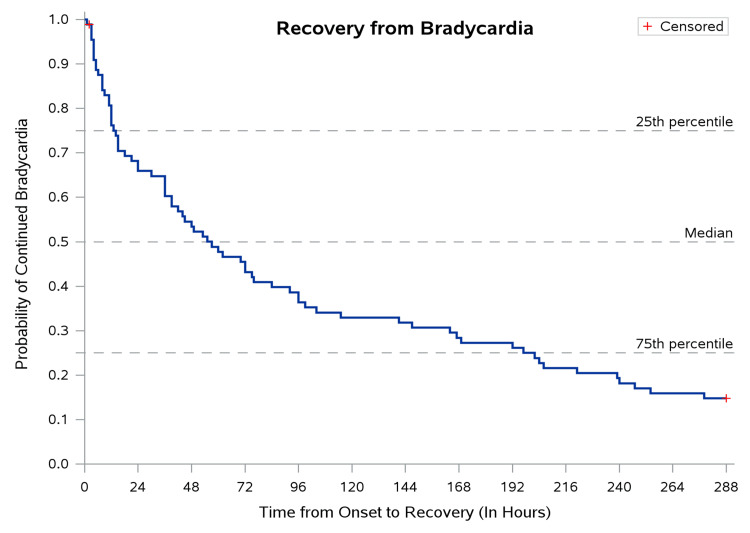
Time to recovery from bradycardia following steroid administration Median time to recovery from bradycardia following steroid administration. Error bars represent the IQR. IQR: interquartile range

The median time to recovery from bradycardia after onset was 57.0 (IQR: 14.0-202.0) hours, as shown in Figure 3.

There was a significant drop in heart rate immediately after the administration of methylprednisolone (p < 0.05) and the first time bradycardia was first noted (p < 0.0001) for each age group.

However, the average drop-in heart rate did not differ significantly across the age groups (p = 0.7). The average heart rate was significantly higher at discharge for each group (p < 0.001), although many did not reach the normal range. As time-to-event data (time to onset of bradycardia and time to recovery) were right-skewed and subject to censoring (children discharged before full recovery), Kaplan-Meier survival curves were used to estimate median time-to-event with interquartile ranges. The non-parametric Mood's two-sample median test was used to compare median times between groups, as it makes no distributional assumptions and is appropriate for heavily skewed survival data. A two-tailed p-value of less than 0.05 was considered statistically significant for all tests.

Based on the Kaplan-Meier curves, the median time to onset of bradycardia after the administration of methylprednisolone is 31 hours, with the interquartile range being 10-80 (Figure 2). Similarly, the median time to recovery from bradycardia after the onset is 57 hours, with the interquartile range being 14-202, as shown in Figure 3.

**Table 3:** Association of heart rate with dose of methylprednisolone and shock Heart rate parameters are compared between children receiving pulse-dose versus conventional-dose methylprednisolone and between those with and without shock. Continuous variables are expressed as mean ± standard deviation and were compared using the Student's t-test. Categorical variables are expressed as number (percentage) and were compared using the chi-square test. A p-value of <0.05 was considered statistically significant (*). HR: heart rate, SD: standard deviation

HR	Number	Overall (n = 89)	Pulse dose (n = 37)	Conventional dose (n = 52)	|t| value	p-value	With shock (n = 16)	Without shock (n = 73)	|t| value	p-value
Bradycardia (number (%))	-	75 (84.3%)	31 (83.9%)	44 (84.6%)	0.01	0.09	14 (87.5%)	61 (83.6%)	0.15	0.7
HR at admission (mean ± SD)	89	133.4 ± 21.5	134.0 ± 17.8	133.1 ± 24.0	0.20	0.8	138.4 ± 14.7	132.3 ± 22.7	1.02	0.3
HR before steroid (mean ± SD)	89	119.0 ± 19.2	120.8 ± 21.6	117.8 ± 17.3	0.74	0.5	122.9 ± 22.0	118.2 ± 18.6	0.90	0.4
HR at bradycardia (mean ± SD)	75	79.7 ± 13.5	74.6 ± 13.3	83.2 ± 12.6	2.84	0.006*	69.9 ± 13.4	81.9 ± 12.5	3.18	0.007*
HR at discharge (mean ± SD)	89	98.5 ± 17.4	93.7 ± 18.3	101.9 ± 16.1	2.24	0.03*	95.4 ± 17.3	99.2 ± 17.5	0.79	0.4

Similarly, although there was no significant difference (p = 0.1) in the proportion of those who developed bradycardia between those who had pulse dose (32, 86.5%) and those who had conventional dose (44, 84.6%) and there was no significant difference in mean heart rate at admission (p = 0.8) or just before methylprednisolone administration (p = 0.5), the mean heart rate of those who had bradycardia and were on pulse dose was significantly lower (p = 0.006) than those on conventional dose and continued to be significantly lower (p = 0.03) at discharge. This dose-severity gradient, where a higher steroid dose produced a lower heart rate nadir despite similar bradycardia incidence, is consistent with a dose-dependent pharmacological effect on cardiac chronotropy and supports a drug-effect explanation over disease-related conduction system involvement.

Similarly, the proportion of children who developed bradycardia did not differ significantly between those who presented with shock (14/16, 87.5%) and those without shock (61/73, 83.6%) (p = 0.4). Mean heart rates at admission (p = 0.3) and prior to steroid initiation (p = 0.4) were comparable between the two groups. However, among children who developed bradycardia, those with shock had significantly lower heart rates at the time of bradycardia onset compared to those without shock (p = 0.007). This difference was no longer statistically significant at discharge (p = 0.4). The distribution of time to onset of bradycardia and time to recovery, stratified by steroid dose and presence of shock, is presented in Table 4.

**Table 4:** Association of time to onset and recovery with dose of methylprednisolone and shock Time to onset and recovery of bradycardia are presented as median with IQR and were compared using Mood's two-sample test; p < 0.05 was considered statistically significant. Time is measured in hours. IQR: interquartile range

	Overall (n = 75)	Pulse dose (n = 31)	Conventional dose (n = 44)	|z| value	p-value	With shock (n = 14)	Without shock (n = 61)	|z| value	p-value
Time to onset (median (IQR))	31.0 (10.0-80.0)	37.0 (15.0-80.0)	21.5 (7.5-82.5)	0.9	0.4	51.0 (10.0-96.0)	27.0 (9.0-75.0)	0.2	0.9
Time to recovery (median (IQR))	57.0 (14.0-202.0)	74.0 (30.0-230.0)	41.5 (11.0-144.0)	0.6	0.5	53.0 (21.0-197.0)	57.0 (13.0-202.0)	0.6	0.6

Overall, the median time taken, with IQR, to the onset of bradycardia after the administration of methylprednisolone was 31.0 (10.0-80.0) hours. However, the time taken for those on conventional dose (21.5 (7.5-82.5) hours) was lower than that of those on pulse dose (37.0 (15.0-80.0) hours), but not significant (p = 0.7). Similarly, the median time taken for those without shock (27.0 (9.0-75.0) hours) was lower than that of those with shock (51.0 (10.0-96.0) hours), but not significant (p = 0.9).

For time to recover from bradycardia after the onset, the overall median time taken, with IQR, was 57.0 (14.0-202.0) hours. However, the time taken for those on conventional dose (41.5 (11.0-144.0) hours) was lower than those on pulse dose (74.0 (30.0-230.0) hours), but not significant (p = 0.3). Similarly, the time taken for those without shock (57.0 (13.0-202.0) hours) was lower than that of those with shock (53.0 (21.0-197.0) hours), but not significant (p = 0.7).

Moreover, none of our patients were symptomatic due to bradycardia. Hence, active interventions were not required. Our patients continued steroid therapy despite bradycardia, and a few were discharged before bradycardia was resolved. This 31-hour median delay is clinically significant, as it falls well outside the immediate post-infusion window and may be misattributed to disease progression if the temporal relationship with methylprednisolone administration is not recognized by the treating clinician.

## Discussion

In this longitudinal study of 89 previously healthy children with MIS-C who received methylprednisolone, an 84.3% prevalence of delayed and sustained sinus bradycardia was observed. This high prevalence was statistically significant and consistent across all pediatric age groups, from infants under one year to school-age children, suggesting an age-independent pharmacological phenomenon rather than a manifestation of the underlying inflammatory disease. The median time to onset of bradycardia after methylprednisolone administration was 31 (IQR: 10-80) hours, and the median time to recovery was 57 (IQR: 14-202) hours. Notably, all 75 children who developed bradycardia were asymptomatic, none required pharmacological intervention, and steroid therapy was continued in all cases without interruption.

While the proportion of children developing bradycardia did not differ significantly between those receiving pulse-dose (30 mg/kg/day; 86.5%) versus conventional-dose (2 mg/kg/day; 84.6%) methylprednisolone (p = 0.1), the heart rate at the bradycardia nadir was significantly lower in the pulse-dose group (74.6 ± 13.3 bpm versus 83.2 ± 12.6 bpm; p = 0.006). This dose-severity gradient is consistent with a dose-dependent pharmacological effect on cardiac chronotropy.

Similarly, although the proportion of children developing bradycardia did not differ significantly between those presenting with and without hemodynamic shock (p = 0.7), the heart rate nadir was significantly (p = 0.007) lower in children presenting with hemodynamic shock (69.9 ± 13.4 bpm) versus those without (81.9 ± 12.5 bpm), suggesting that pre-existing hemodynamic compromise may potentiate the bradycardic response. That is, cardiac involvement due to the disease itself could explain the bradycardia. However, our patients continued to improve clinically, and none of them required any pharmacological interventions; their heart rate gradually became normal, suggesting that the observed bradycardia is independent of the disease. Moreover, as mentioned above, a significantly lower heart rate for those on pulse dose compared to the conventional dose suggests that the bradycardia observed here is due to the drug effect rather than the disease. Therefore, clinically, the bradycardia seems to be independent of the disease. However, our study did not have a non-steroid control group, and multivariate analysis was not performed to prove this statistically.

The scientific rationale for this study is rooted in a diagnostic dilemma that is uniquely challenging in MIS-C. Unlike in other inflammatory conditions where steroid-induced bradycardia occurs in the setting of a preserved cardiac substrate, children with MIS-C have intrinsic cardiac disease at the time of treatment. Cardiac involvement in MIS-C occurs in up to 80% of cases and encompasses myocarditis, ventricular dysfunction, coronary artery dilation, pericarditis, and primary arrhythmias [15]. A recent systematic review and meta-analysis encompassing 84 studies and 4,778 patients confirmed that cardiovascular manifestations, including reduced ejection fraction, diastolic dysfunction, and coronary abnormalities, begin during the acute phase and may persist in a subset of patients well beyond discharge [16]. In this context, the emergence of bradycardia in a child receiving methylprednisolone for MIS-C presents a profound clinical interpretation problem. Bradycardia may represent (a) a pharmacological side effect of methylprednisolone, which is the hypothesis supported by this study, and (b) a manifestation of MIS-C-associated myocarditis with direct involvement of the cardiac conduction system.

Distinguishing the above two has important clinical consequences. Misattributing drug-induced, self-limiting bradycardia to disease-related cardiac deterioration may prompt unnecessary escalation of cardiac investigations, including repeated echocardiography, 12-lead electrocardiography, and extended ICU monitoring, as well as unnecessary interruption of steroid therapy, which remains the cornerstone of MIS-C management [17]. This is particularly relevant in resource-limited settings like ours, where the opportunity cost of unnecessary investigations is high.

Therefore, to be able to distinguish receptor-level pharmacodynamic modulation from acute disease progression, we examined the time of onset of and time of recovery from bradycardia and the association with dose and with the presence of hemodynamic shock. We also compared the presence of bradycardia across age groups.

The 84.3% prevalence of bradycardia in our cohort is consistent with, and adds important context to, existing literature on corticosteroid-induced bradycardia across pediatric inflammatory conditions. Prior to this study, the most systematic evidence came from two pediatric conditions. In children with acute lymphoblastic leukemia (ALL) receiving induction chemotherapy containing corticosteroids, Duffy et al. reported bradycardia in up to 98% of patients, with clinically significant bradycardia in 59%[1]. Similarly, in severe Kawasaki disease, Nagakura et al. demonstrated a bradycardia prevalence of 79.1% among children receiving IVIG plus prednisolone, compared to only 7.1% in those receiving IVIG alone, thereby providing one of the most compelling arguments for corticosteroid pharmacology, rather than underlying disease, as the primary driver of bradycardia [2].

Similar observations were documented in rheumatologic disorders [18], nephrotic syndrome, and juvenile arthritis [19,20], all of which resolved spontaneously. Moreover, recent pediatric case reports confirm that bradycardia occurs even with low-dose steroid regimens [21,22].

The present study advances substantially beyond these earlier reports in three respects. First, this is among the largest single-center cohorts specifically examining methylprednisolone-associated bradycardia in MIS-C (n = 89), substantially larger than prior MIS-C case reports and series on this topic [7,8,23]. Second, the use of Kaplan-Meier survival analysis provides the first systematic temporal characterization of this phenomenon in MIS-C, quantifying a median onset of 31 hours and a median recovery of 57 hours, benchmarks that give clinicians a practical reference window for distinguishing drug effect from disease progression. Third, this is the first study to examine whether steroid dose and hemodynamic shock independently modify the severity of the bradycardic response in MIS-C, finding that, while incidence was similar across groups, the heart rate nadir was significantly lower in both the pulse-dose and shock subgroups.

In the non-pediatric literature, Stroeder et al. systematically reviewed corticosteroid-induced bradycardia and found that symptomatic and asymptomatic episodes both resolved without intervention in 3-10 days [24]. Our median recovery time of 57 hours falls within the lower end of this range and is consistent with prior pediatric reports where bradycardia resolved within 24-36 hours after drug cessation [25]. Importantly, in our study, resolution occurred without drug discontinuation, further reinforcing the benign and self-limiting nature of this association.

The precise mechanism of methylprednisolone-associated bradycardia in children remains an area of active investigation, and several non-mutually exclusive pathways have been proposed. First, high-dose glucocorticoids may exert direct electrophysiological effects on cardiac ion channels, particularly by reducing calcium influx through L-type calcium channels and altering potassium channel conductance, thereby slowing sinoatrial node automaticity [24,26]. Second, pulse corticosteroids may induce changes in sodium and water physiology, leading to plasma volume expansion and reflex activation of low-pressure baroreceptors, thereby augmenting vagal tone and slowing the sinus rate [24]. Third, glucocorticoid receptor-mediated downregulation of cardiac beta-adrenergic receptors may reduce sympathetic drive to the heart, resulting in relative bradycardia [24,27]. Animal studies have confirmed that high-dose methylprednisolone has significant direct myocardial membrane effects and alters cardiovascular sensitivity to catecholamines [26].

Our study cannot determine which mechanism predominated in individual patients, and future studies incorporating biomarker profiling, cardiac MRI, and electrophysiological assessment are needed.

This study has several notable strengths. First, with 89 eligible patients from a single tertiary referral center in central Kerala, India, this is among the largest cohort studies examining methylprednisolone-associated bradycardia specifically in MIS-C. The relatively large sample size for a single-center study allows subgroup analyses by steroid dose and shock status with adequate statistical power. Second, the strict exclusion criteria, specifically excluding children with prior steroid exposure within four weeks and those with pre-existing cardiac conditions, substantially reduced confounding and isolated the effect of methylprednisolone in a previously healthy cardiac substrate. Third, the availability of second-hourly heart rate documentation from continuous bedside cardiac monitoring, extracted systematically from nursing records, provided dense and reliable temporal data that enabled Kaplan-Meier survival analysis of time-to-event endpoints. Fourth, structured outpatient follow-up at one week, two weeks, and one month allowed characterization of recovery trajectories beyond the inpatient period. Fifth, the use of the WHO-UMC causality assessment scale to classify the association as "possible" adds a structured pharmacovigilance framework that is consistent with the level of evidence available and is transparent about the limits of causal inference [10]. Finally, this study was conducted in a resource-limited setting in South Asia, contributing geographically diverse data to the predominantly high-income country literature on MIS-C.

This study has several important limitations that must be considered when interpreting its findings. First, and most critically, the absence of a non-steroid comparison group (i.e., children with MIS-C who did not receive methylprednisolone) means that it is not possible to definitively attribute bradycardia to the drug rather than the underlying disease. MIS-C itself causes myocarditis and conduction system involvement, which could independently precipitate bradycardia, and this potential confounding factor cannot be fully excluded based on temporal association alone. Second, the study relied on bivariate analysis, Student's t-test, chi-square, and Kaplan-Meier with Mood's median test, and did not include multivariate logistic or Cox regression analysis. As a result, independent predictors of bradycardia severity cannot be rigorously identified, and the effect of potential confounders such as baseline inflammatory marker levels, echocardiographic parameters, or concomitant IVIG use was not formally adjusted for. Hence, the independent contribution of methylprednisolone to the observed bradycardia cannot be statistically isolated from MIS-C-related conduction system involvement. Third, electrolyte levels, blood pressure trends, and vagal tone markers were not systematically analyzed, limiting the mechanistic interpretation of the observed bradycardia. Fourth, the study population consisted entirely of previously healthy children without comorbidities, limiting generalizability to immunocompromised or chronically ill children. Future prospective multicenter studies incorporating continuous ambulatory heart rate monitoring, serial electrolyte profiling, echocardiographic assessment, autonomic function testing, and a concurrent non-steroid comparison arm are needed to establish definitive causality, identify independent predictors of bradycardia severity, and develop evidence-based monitoring protocols for steroid use in MIS-C.

This study makes several distinct and cumulative contributions to the scientific literature on MIS-C and corticosteroid pharmacology in children. First, it provides the first systematic quantification of the incidence, temporal onset, duration, and recovery profile of bradycardia in MIS-C, specifically, using robust time-to-event methodology rather than anecdotal case reporting. The Kaplan-Meier estimates of median onset (31 hours) and recovery (57 hours) provide clinicians with empirically grounded temporal reference points that are absent from all prior MIS-C literature. Second, it is the first study to demonstrate that while overall bradycardia incidence is similar across steroid dose groups and shock subgroups, the depth of heart rate reduction (nadir) is significantly greater in pulse-dose and shock patients, offering a novel, clinically actionable risk stratification framework. Third, by documenting 84.3% bradycardia prevalence across all age groups, this study establishes that the bradycardic effect is an age-independent class effect of methylprednisolone, not a disease-specific complication of MIS-C, and thereby positions it within the broader literature on corticosteroid-induced cardiac chronotropy across pediatric conditions, including ALL [1] and Kawasaki disease [2]. Fourth, the pharmacovigilance framework applied, with WHO-UMC causality classification, is a methodologically structured approach to adverse drug reaction reporting that is underutilized in the pediatric infectious disease literature and provides a replicable model for future studies. Fifth, this study addresses a significant gap in the MIS-C outcome literature that is predominantly from high-income countries by generating evidence from a resource-limited South Asian tertiary center, where healthcare resource optimization is of clinical and policy relevance. Collectively, these contributions build the evidentiary foundation for the development of evidence-based clinical monitoring protocols for steroid use in MIS-C and create a compelling rationale for prospective, multicenter trials with continuous ambulatory monitoring, electrolyte profiling, and autonomic function assessment.

## Conclusions

The findings of our study have direct and actionable implications for the clinical management of MIS-C in both inpatient and outpatient settings. Bradycardia is a common occurrence, observed in 84.3% of our cohort, following methylprednisolone in MIS-C, regardless of age, dose, or the presence of shock. Clinicians who recognize the characteristic temporal profile of this association, delayed onset at a median of 31 (IQR: 10-80) hours, sustained duration of approximately 57 hours, asymptomatic nature, and spontaneous resolution, are well-positioned to avoid unnecessary interventions when this pattern is observed in an otherwise clinically stable child.

In many clinical centers, the detection of bradycardia in a child with MIS-C, a condition strongly associated with myocarditis, reflexively triggers an escalation of cardiac investigations, including repeat echocardiography, 12-lead ECG, and extended ICU monitoring. Our data suggest that in children who develop asymptomatic bradycardia within the expected temporal window after methylprednisolone, where hemodynamic status is otherwise stable, this pattern is far more likely to represent a pharmacological side effect rather than disease deterioration. Accordingly, the current evidence does not support automatic interruption of steroid therapy or escalation of intensive care in this setting. This conclusion must be tempered with the important caveat that in children with more severe presentations, particularly those with shock, closer monitoring is warranted, given the significantly lower heart rate nadir observed in these subgroups. These high-risk children warrant heightened vigilance and individualized clinical judgment. The outpatient implications are also significant. Given that bradycardia persisted beyond discharge in a subset of children, structured outpatient heart rate monitoring is recommended for at least two to four weeks following discharge. Risk-stratified outpatient cardiac surveillance, rather than a uniform approach for all patients, is the most rational management framework.
