# Correction: Methylprednisolone-Induced Delayed and Sustained Bradycardia in Multisystem Inflammatory Syndrome in Children

**DOI:** 10.7759/cureus.c458

**Published:** 2026-07-02

**Authors:** Sujin M Thomas, Jijo J John, Keerthy Kurian, Alice David, Aby Dany Varghese, Midhuna TV, Jomon V Thomas

**Affiliations:** 1 Pediatric Medicine, Believers Church Medical College, Thiruvalla, IND; 2 Pediatric Medicine, Believers Church Medical College Hospital, Thiruvalla, IND; 3 Medical Research, Believers Church Medical College Hospital, Thiruvalla, IND; 4 Pediatric Medicine, Balagangadharanatha Swamiji (BGS) Medical College and Hospital, Adichunchanagiri University, Bangalore, IND

This article has been corrected to fix several typos throughout the article, as well as incorrect data in Table 2. The corrections are as follows:

Statistical Methods section:

- "Complete heart rate data at all time points were available for 87 of the 89 included patients." corrected to "Complete heart rate data at all time points were available for all 89 included patients."
- "In two patients discharged before bradycardia resolution, their time-to-recovery was treated as censored observations in the Kaplan-Meier analysis, consistent with standard survival analysis methodology" corrected to "Patients discharged before resolution of bradycardia was treated as censored observations."

Results section:

- An incorrect reference to Figure 2 was removed.
- An incorrect reference to Figure 3 was corrected to reference Figure 2.
- An incorrect reference to Table was corrected to reference Figure 3.
- In Table 2, the values entered in the 'Number' column have been corrected.

Discussion section:

- "76" has been corrected to "75" in the following sentence: "Notably, all 76 children who developed bradycardia were asymptomatic, none required pharmacological intervention, and steroid therapy was continued in all cases without interruption."

